# Exploration of Potential Integrated Models of N6-Methyladenosine Immunity in Systemic Lupus Erythematosus by Bioinformatic Analyses

**DOI:** 10.3389/fimmu.2021.752736

**Published:** 2022-02-07

**Authors:** Xingwang Zhao, Lan Ge, Juan Wang, Zhiqiang Song, Bing Ni, Xiaochong He, Zhihua Ruan, Yi You

**Affiliations:** ^1^ Department of Dermatology, Southwest Hospital, Army Medical University (Third Military Medical University), Chongqing, China; ^2^ Department of Pathophysiology, College of High Altitude Military Medicine, Army Medical University (Third Military Medical University), Chongqing, China; ^3^ Department of Nursing Administration, Faculty of Nursing, Army Medical University (Third Military Medical University), Chongqing, China; ^4^ Department of Oncology and Southwest Cancer Center, Southwest Hospital, Army Medical University (Third Military Medical University), Chongqing, China

**Keywords:** systemic lupus erythematosus, m6A, immune, immune cell infiltrations, bioinformatic analyses

## Abstract

Systemic lupus erythematosus (SLE) is a prototypical systemic autoimmune disease of unknown etiology. The epigenetic regulation of N6-methyladenosine (m6A) modification in immunity is emerging. However, few studies have focused on SLE and m6A immune regulation. In this study, we aimed to explore a potential integrated model of m6A immunity in SLE. The models were constructed based on RNA-seq data of SLE. A consensus clustering algorithm was applied to reveal the m6A-immune signature using principal component analysis (PCA). Univariate and multivariate Cox regression analyses and Kaplan–Meier analysis were used to evaluate diagnostic differences between groups. The effects of m6A immune-related characteristics were investigated, including risk evaluation of m6A immune phenotype-related characteristics, immune cell infiltration profiles, diagnostic value, and enrichment pathways. CIBERSORT, ESTIMATE, and single-sample gene set enrichment analysis (ssGSEA) were used to evaluate the relative immune cell infiltrations (ICIs) of the samples. Conventional bioinformatics methods were used to identify key m6A regulators, pathways, gene modules, and the coexpression network of SLE. In summary, our study revealed that IGFBP3 (as a key m6A regulator) and two pivotal immune genes (CD14 and IDO1) may aid in the diagnosis and treatment of SLE. The potential integrated models of m6A immunity that we developed could guide clinical management and may contribute to the development of personalized immunotherapy strategies.

## Introduction

Systemic lupus erythematosus (SLE) is a complex, multisystem, and chronic-relapsing immune disorder with diverse clinical manifestations and significant morbidity and mortality (1). The pathogenesis of SLE has not been fully elucidated. Thus, it is necessary to characterize this complex disease by analyzing high-throughput sequencing data.

Multiple epigenetic processes, including N6-methyladenosine (m6A) RNA modification, have been shown to play a major role in the pathogenesis of SLE (2, 3). These processes are associated with the genetic risk of SLE, and their interaction in T cells is thought to contribute to SLE development (4, 5). m6A modification is involved in various biological processes (mRNA splicing, stability, translation, etc.), which exist in most RNAs and organisms (6). m6A is regulated by m6A reader proteins (“readers”), demethylases (“erasers”), and methyltransferases (“writers”), which are the most abundant epigenetic modifications of mRNAs (7–9). The dysregulated immune response plays a significant role in SLE and immunological pathogenesis, including innate and adaptive immunity (10). Moreover, several studies have shown that the dysfunction of multiple immune cells, such as monocytes, dendritic cells, neutrophils, T cells, and B cells, plays a crucial role in the pathogenesis of SLE (11–14). However, the function of m6A in the immune system of a person with SLE remains unknown.

In this study, we intended to screen promising biomarkers for the diagnosis and treatment of SLE. We proposed and produced different subtype classifications of m6A immune profiles from independent datasets. Multiple algorithms were adopted to explore the m6A-immune characteristic classification of SLE and to guide us in improving the diagnosis and efficacy of immunotherapy. Associations of m6A or ICI score and diagnosis were analyzed across several different datasets. The m6A immune patterns were systematically evaluated, and this evaluation revealed the potential role of the m6A immune landscape in SLE.

## Materials and Methods

### Data Collection and Processing

Our study extracted data from the Gene Expression Omnibus (GEO) database (https://www.ncbi.nlm.nih.gov/geo/). Normalized microarray gene expression data of GSE49454 (whole blood, **Supplementary Tables S1, S2**), GSE61635 (whole blood, **Supplementary Table S3**), GSE110169 (whole blood, **Supplementary Tables S4, S5**), and GSE72509 (whole blood, **Supplementary Table S6**) were used as training sets to screen key m6A regulators. Out of these four datasets, only diagnostic data for GSE49454 were available. GSE50772 (peripheral blood mononuclear cells, **Supplementary Table S7**), GSE81622 (peripheral blood mononuclear cells, **Supplementary Table S8**), GSE122459 (peripheral blood mononuclear cells, **Supplementary Table S9**), GSE20864 (whole blood, **Supplementary Table S10**), GSE39088 (whole blood, **Supplementary Table S11**), and GSE156751 (plasmablast B cells, naive B cells, and memory B cells, **Supplementary Table S12**) were available from the GEO database as verification sets. The ComBat function of the R software package sva was selected to eliminate batch differences (15). The basic information of the datasets selected is shown in **Supplementary Table S13**.

### Identifying M6A Regulators and Enrichment Analysis

To screen m6A regulators, all samples from 10 datasets were analyzed using the empirical Bayesian method of the limma package of R. The criterion for screening m6A regulators was an adjusted p-value <0.05. The common m6A-mediated genes overlapped according to a Venn plot. Enrichment analyses of the Gene Ontology (GO) and Kyoto Encyclopedia of Genes and Genomes (KEGG) pathways were conducted using Metascape (http://metascape.org/gp/index.html#/main/step1) and FunRich (functional enrichment analysis tool) (http://www.funrich.org/), respectively. The coexpression networks of the m6A regulators were constructed in GENEMANIA software (http://genemania.org/search/).

### Identification of Potential M6A Regulators Associated With SLE

The comparative toxicogenomics database (CTD, http://ctdbase.org/) integrates information, including gene–disease relationships, to explore the underlying pathogenesis of diseases (16). The association between potential crucial m6A genes and SLE risk was analyzed using the CTD data.

### Transcription Factors and MicroRNAs Interact With M6A Regulator Analysis

The interaction network of microRNAs (miRNAs) and transcription factors (TFs) associated with the key m6A regulators was predicted by Networkanalyst (https://www.networkanalyst.ca/).

### Correlation Analysis

The “corrplot” R (17) package was used to perform the correlation analysis of m6A regulators or immune cell infiltration. When the correlation coefficient is >0.5, the correlation is positive. When the correlation coefficient is <0.5, the correlation is negative.

### Consensus Clustering of the M6A Model

RNA-seq data of 23 m6A regulators, including 8 “writers” (METTL14, METTL3, WTAP, METTL16, VIRMA, RBM15B, ZC3H13, and RBM15), 13 “readers” (YTHDF1, HNRNPA2B1, YTHDF2, YTHDC1, IGF2BP1, RBMX, HNRNPC, LRPPRC, YTHDC2, IGF2BP2, IGF2BP3, YTHDF3, and FMR1), and 2 “erasers” (ALKBH5 and FTO), were obtained from the datasets. Least absolute shrinkage and selection operator (LASSO) regression was used to construct the m6A diagnostic signature by feature selection and dimension reduction (18). Cross-validation was used for lambda parameter tuning. The cutoff value was decided by rank statistics. Unsupervised clustering analysis was used to identify different m6A modification patterns. The cluster numbers and robustness were evaluated as described in a previous study (19). The R package “ConsensusClusterPlus” was used for classification (20). PCA was used to validate the signature score. The m6A gene expression and immune cell infiltration abundance scores among the distinct modification patterns were compared. The enrichment scores of immune cell abundance and immune reactivity in healthy and SLE samples were compared by Wilcoxon assay.

### Consensus Clustering of the ICIscore Model

In this study, the “ConsensusClusterPlus” R (20) package was used to cluster samples based on gene expression, and 1,000 iterations were performed to ensure the stability of classification. We used the “CIBERSORT” package R (21) to quantify the levels of infiltration of different immune cells in different datasets. Different immune cell subtypes were grouped and utilized to compare the discrepancies of different clusters. Dimension reduction in ICI gene clusters was based on the Boruta algorithm and the signature score using PCA. ESTIMATE and CIBERSORT algorithms were used for the analysis of immune status, such as immune and stromal scores (21, 22). For the ICI score, the following method was used (23): ICI score = ∑PCI_A_ − ∑PCI_B._

### Univariate and Multivariate Cox Analyses

We downloaded gene expression and clinical characteristic data to perform univariate and multivariate Cox analyses to identify whether the expression of m6A regulators or immune genes was related to the diagnosis of SLE patients. Several clinicopathological parameters were included. The m6A regulators or immune genes were identified by univariate logistic regression (cutoff criterion: p-value <0.05). Multivariate logistical regression was applied to verify m6A regulators related to the SLE classifier.

### Construction of the Diagnostic Gene Signature

Gene signature univariate Cox regression analysis (p < 0.001 was considered statistically significant) and LASSO‐penalized Cox regression analysis (24) were used to verify the diagnostic gene signature. Diagnostic analysis of m6A immune-related genes was identified using the “survminer” and “survival” R packages (p < 0.05 was statistically significant) (25). In particular, the “survminer” R package was used to divide patients into high‐ and low‐risk groups according to the optimal cutoff value. The “survivalROC” (26) R package was used to determine the time‐dependent diagnostic value of the gene signature (log‐rank p < 0.05 was considered significant).

### Immune Gene Analysis and Risk Model Construction

The list of infiltrating immune cell gene sets was derived from a previous study (19), and the immune gene sets were obtained from the ImmPort database (http://www.immport.org) (27). Univariate Cox regression analysis and LASSO-penalized Cox regression analysis were applied to verify the diagnostic signature in the training dataset. The risk score was calculated as follows: risk score = SExpi*bi. Expi represents each gene expression, and bi represents the coefficient of each gene.

### Weighted Gene Coexpression Network Analysis

Weighted gene coexpression network analysis (WGCNA) was performed using the WGCNA R package (28) to build a gene coexpression network, and key genes related to the significant modules were identified. Gene modules with |correlation coefficient| >0.5 were considered strongly correlated modules, and significantly diagnosis-associated hub immune genes were selected for further analysis (p < 0.05, log-rank test). Strong diagnosis-correlated gene modules were used as an input in the LASSO regression analysis.

### GO and KEGG Analyses

The GO (29) R package was used to display the results of the GO and KEGG analyses. All differentially expressed m6A immune genes were selected for GO and KEGG pathway analyses. GO analysis covers three domains: biological process (BP), cellular component (CC), and molecular function (MF). The importance of the GO and KEGG pathways was identified [false discovery rate (FDR) <0.05 denotes the significance of the p value].

### Gene Set Variation Analysis

The Gene Set Variation Analysis (GSVA) package in R (30) was used to evaluate the most significantly enriched molecular pathways. The gene sets of “c2.cp.kegg.v7.0.symbols” and “h.all.v7.0.symbols’ were downloaded from the Molecular Signatures Database (MSigDB, version 6.0) database for running GSVA analysis. Differential analysis of the enrichment scores of KEGG pathways between SLE patients and healthy controls was performed using the R package limma (31).

### Gene Set Enrichment Analysis and Single-Sample GSEA

The significantly enriched pathways in samples with ICI score-high or ICI score-low were identified by GSEA according to the MSigDB. The number of infiltrating immune cells and the activity of the immune response were estimated by ssGSEA, and the enrichment fraction represents the absolute enrichment degree of a gene set in each sample (32).

### Statistical Analysis

R software (https://www.r-project.org/, version 4.1) and corresponding R packages (http://www.bioconductor.org/) were utilized for statistical analysis. The results are presented as the mean ± SD (p < 0.05 indicated statistical significance). Student’s t-test was applied for continuous variable analysis. Spearman’s correlation was adopted to analyze correlations. Kaplan–Meier analysis was performed, and diagnostic values were compared by the log-rank test.

## Results

### Identification of m6A Regulators Between SLE Patients and Healthy Controls

**Figure 1:**
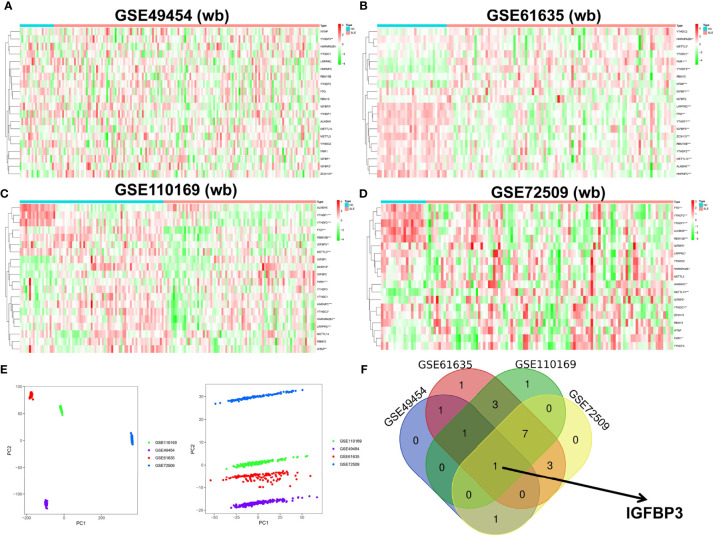
Identification of the key m6A regulators in the four GEO datasets. **(A–C)** Heatmap showing the expression status of m6A regulators between SLE and healthy controls in **(A)** GSE49454, including 4 m6A regulators; **(B)** GSE61635, including 17 m6A regulators; **(C)** GSE110169, including 13 m6A regulators; and **(D)** GSE72509, including 12 m6A regulators. Green: low expression level; red: high expression level. **(E)** Principal component analysis for the expression of genes to distinguish GEO cohorts of SLE (GSE49454, GSE61635, GSE110169, and GSE72509). **(F)** Venn diagram to identify m6A regulators between SLE and healthy samples. *p < 0.05, **p < 0.01, ***p < 0.001.

Based on the high-throughput data analysis, four whole-blood datasets were used as training sets to screen the key m6A regulators based on the expression levels of m6A genes. The results showed that 4 m6A regulators were significantly differentially expressed in the GSE49454 dataset (**Figure 1A**), 17 m6A regulators were significantly differentially expressed in the GSE61635 dataset (**Figure 1B**), 13 m6A regulators were significantly differentially expressed in the GSE110169 dataset (**Figure 1C**), and 12 m6A regulators were significantly differentially expressed in the GSE72509 dataset (**Figure 1D**). The principal component analysis of GSE49454, GSE61635, GSE110169 and GSE72509 datasets were performed by unsupervised clustering based on the expression of genes (**Figure 1E**). The Venn diagram showed that IGFBP3 overlapped in the four datasets (**Figure 1F**), which suggests that it plays an important role in SLE patients.

### Functional Pathway, Gene Network, and Relationship to SLE Analysis of m6A Regulators

**Figure 2:**
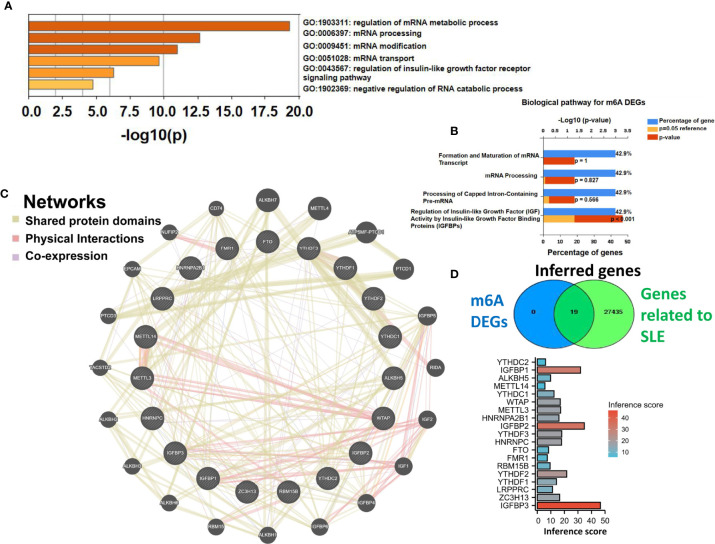
GO, KEGG, gene–gene interaction network analyses, and relationship to SLE of m6A regulators. **(A)** Bar plot showing the GO analysis of the 19 identified m6A regulators based on Metascape online. **(B)** KEGG analysis of biological pathways (ranked by p-value). **(C)** The gene–gene interaction network of the 19 identified m6A regulators constructed by GENEMANIA software. **(D)** Relationship to SLE of the 19 identified m6A regulators based on the CTD database.

GO and KEGG pathway analyses revealed the functions of the 19 m6A regulators. The GO results showed that they were mainly enriched in the regulation of mRNA metabolic processes, mRNA processing, and related processes (**Figure 2A**). The KEGG results showed that these regulators were mainly enriched in the regulation of insulin-like growth factor (IGF) activity by the insulin-like growth factor-binding protein (IGFBP) pathway (**Figure 2B**). In addition, the gene–gene interaction network for 19 m6A regulators was constructed by the GeneMANIA database. The hub nodes represent genes that were significantly correlated with 19 m6A regulators (**Figure 2C**). CTD was employed to explore the interaction between potential crucial genes and SLE. Inference scores in CTD reflected the association between disease and 19 m6A regulators. The interaction results showed that IGFBP3 has a higher inference score with SLE (**Figure 2D**).

### Identification of IGFBP3 and GSVA Analysis

**Figure 3:**
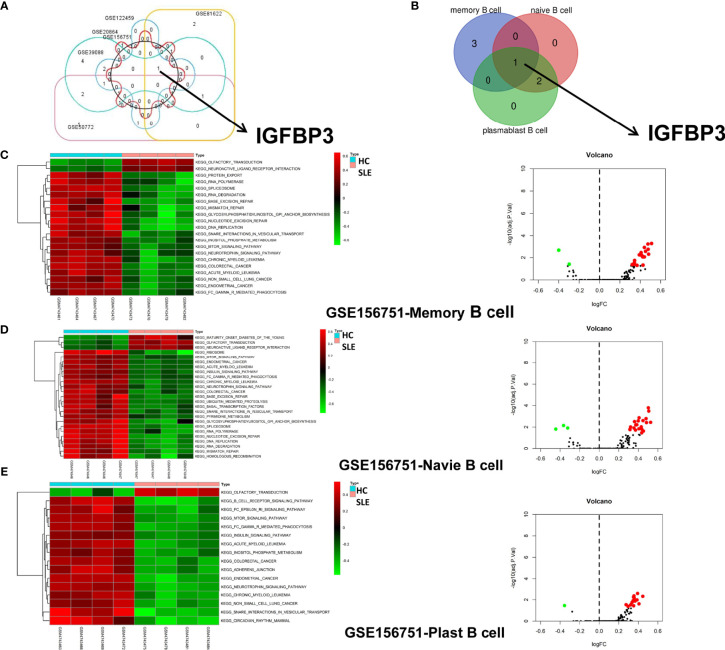
Identification of IGFBP3 as a key m6A target and GSVA analysis. **(A)** Except for GSE50772, the five verification datasets share IGFBP3 overlap. **(B)** The significant differential expression of m6A regulators was identified from three subsets of the GSE156751 dataset based on an adjusted p-value <0.05. The three subsets share IGFBP3 overlapping. **(C–E)** Heatmap and volcano plots illustrating the enrichment scores of differentially enriched molecular pathways evaluated by GSVA analysis between SLE patients and healthy controls (red: high enrichment scores; green: low enrichment scores).

To verify whether IGFBP3 could act as a key target in SLE, based on high-throughput analysis, six datasets were selected as verification sets to screen significantly differentially expressed m6A regulators after the ChIP results were normalized. Among the three PBMC datasets, nine, six, and five m6A regulators were significantly differentially expressed in the GSE50772 dataset (**Supplementary Figure S1A**), the GSE81622 dataset (**Supplementary Figure S1B**), and the GSE122459 dataset (**Supplementary Figure S1C**), respectively. In addition, the m6A regulators in two whole-blood microarray datasets were screened. Four and 15 m6A regulators were significantly differentially expressed in the GSE20864 dataset (**Supplementary Figure S1D**) and the GSE39088 dataset (**Supplementary Figure S1E**), respectively. Four m6A regulators were significantly differentially expressed in the B-cell dataset of GSE156751 (**Supplementary Figure S1E**). The Venn diagram showed that IGFBP3 overlapped in the six verification datasets (**Figure 3A**). Furthermore, IGFBP3 overlapped in the three subsets [GSE156751 (plasmablast B cells), GSE156751 (memory B cells), GSE156751 (naive B cells)] of the GSE156751 dataset (**Figure 3B**). These results also indicated that IGFBP3 could act as a key target in B cells to further explore the detailed molecular mechanism of SLE. Then, GSVA was applied to explore the molecular pathways and underlying mechanisms in B-cell subsets between SLE patients and healthy controls. The top differentially enriched molecular pathways were identified (**Figures 3C–E**). The results showed that olfactory transduction and neuroactive ligand receptor interaction pathways were positively correlated with SLE. DNA replication, RNA polymerase, neurotrophin signaling, spliceosome, etc. pathways were negatively correlated with SLE.

### Univariate and Multivariate Cox Regression Model Analyses of M6A Risk Genes and Construction of a TF–miRNA Network

**Figure 4:**
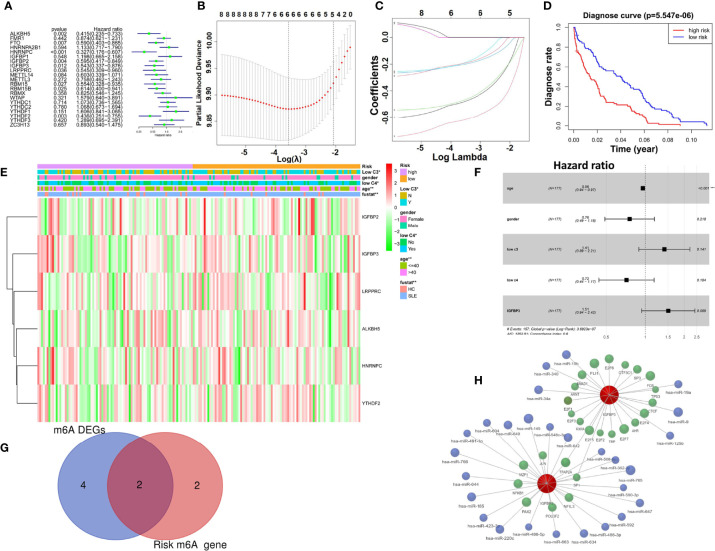
The diagnostic signature can distinguish healthy and SLE samples, and a TF–miRNA network of m6A regulators was constructed in GSE49454. **(A)** Univariate logistic regression demonstrated the relationship between m6A regulators and diagnosis. **(B)** LASSO coefficient profiles of m6A regulators. **(C)** Tenfold cross-validation for tuning parameter selection in the LASSO regression. **(D)** KM plots presenting diagnoses in the high- and low-risk sets. **(E)** Heatmap for the connections between clinicopathologic features and risk factors. **(F)** Multivariate Cox regression model analysis, which included the factors of age, sex, low c3, low c4, and IGF2BP3 in the GSE49454 dataset. **(G)** Venn diagram to identify differentially expressed m6A risk genes. **(H)** TF–miRNA coregulatory network of two m6A regulators (pink: m6A risk genes; blue: miRNA; and green: TF genes). *p < 0.05;**p < 0.01;****p < 0.001.

Univariate Cox regression analysis was used to identify diagnosis-related m6A regulators. Seven genes (HNRNPA2B1, IGFBP1, WTAP, YTHDC1, YTHDC2, YTHDF1, and YTHDF3) were related to increased risk (HRs > 1), including 14 protective genes with HRs <1 (**Figure 4A**). LASSO Cox regression analysis constructed the signature according to the optimum λ value (**Figures 4B, C** **)**. Receiver operating characteristic (ROC) analysis was used to evaluate the sensitivity and specificity for the diagnostic model between the low- and high-risk groups (**Figure 4D**). Furthermore, a heatmap of clinical features for the GSE49454 cohort is shown (**Figure 4E**), and the diagnostic status, low c3, low c4, and age of patients were diversely distributed between the low- and high-risk subgroups (p < 0.05). Meanwhile, we performed multivariate Cox analysis of IGFBP3 for some variables (age, sex, low c3, and low c4) in GSE49454. The multivariate Cox analysis indicated that IGFBP3 (HR = 1.51, p = 0.0089, 95% CI = 0.94–2.42) and age (HR = 0.96, p < 0.001, 95% CI = 0.94–0.97) were more accurate than the other factors (**Figure 4F**). Next, we obtained two m6A risk genes from the intersection of the six m6A risk model genes between the low- and high-risk subgroups and four m6A regulators in GSE49454 (**Figure 4G**). For the two common m6A risk genes (IGFBP2 and IGFBP3), a TF–miRNA gene interaction graph was built using NetworkAnalyst (**Figure 4H**). The network contains a total of 26 TF genes and 27 miRNAs. This interaction might be the reason for regulating the expression of the m6A risk model genes.

### Consensus Clustering of M6A Subtypes With Clinical Features

**Figure 5:**
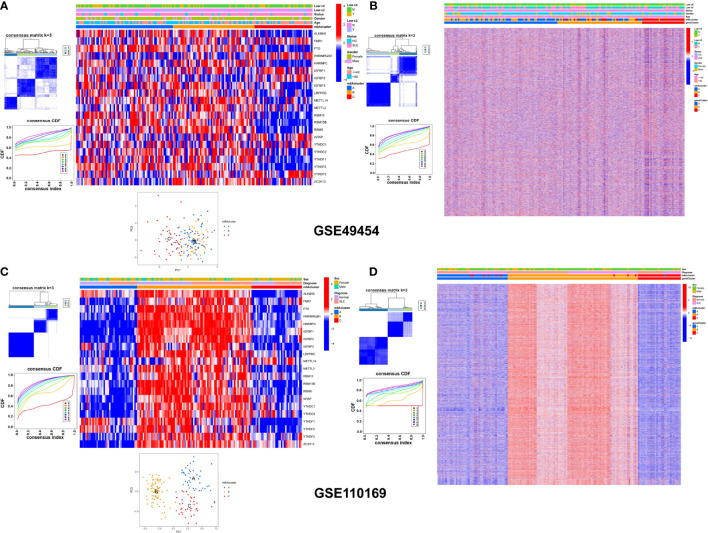
Unsupervised clustering of cohorts based on the genes and m6A clusters. **(A–D)** Consensus clustering classified SLE into three gene clusters and three m6A clusters in the GSE49454 **(A, B)** and GSE110169 **(C, D)** datasets. The upper columns consist of age, sex, low c3, low c4, and m6A clusters of SLE.

Consensus cluster analysis was used to develop the molecular classification of SLE patients according to gene expression. All SLE samples were initially divided into different k (k = 2–9) groups. The cumulative distribution function (CDF) curves showed that the optimal number of clusters was k = 3. Consensus clustering was independently conducted in the GSE49454 (**Figures 5A, B** **)** and GSE110169 (**Figures 5C, D** **)** datasets, as shown in the heatmap, in which SLE patients were categorized into three m6A and gene clusters. The two-dimensional principal component plot showed that the clusters of samples were separated from each other (**Figures 5A, C** **)**.

### Characteristics Analysis of M6A and Gene Clusters

**Figure 6:**
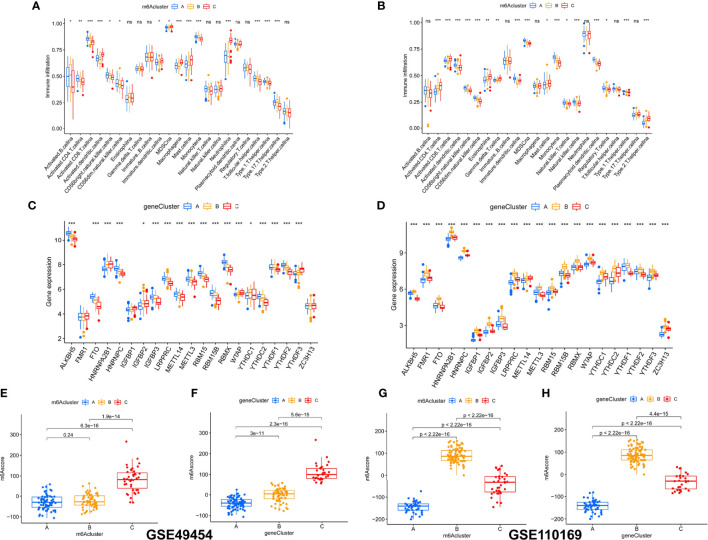
Comparison of the differences in immune cells, m6A regulators, and the m6A score in unsupervised clustering of cohorts. **(A–D)** Comparison of the difference of 23 types of immune cells and 21 m6A regulators on the m6A and gene clusters in the GSE49454 and GSE110169 cohorts. **(E–H)** Comparison of the differences in m6A score on molecular subtypes (gene clusters and m6A clusters) in GSE49454 and GSE110169 datasets. p-values are shown as *p < 0.05; **p < 0.01; ***p < 0.001. ns, not significant.

Infiltrating immune cells were detected to understand the differences in the characteristics of immune infiltration among m6A clusters. Immunocytes differed in GSE49454 and GSE110169. Activated.CD8.T.cellna, Activated.dendritic.cellna, macrophagena, type.1.T.helper.cellna, T.follicular.helper.cellna, mast.cellna, monocytena, neutrophilna, and type.17.T.helper.cellna were significantly different in GSE49454 (**Figure 6A**), while Activated.CD4.T.cellna, Activated.CD8.T.cellna, Activated.dendritic.cellna, immature.B.cellna, immature.dendritic.cellna, CD56bright.natural.killer.cellna, mast.cellna, natural.killer.cellna, plasmacytoid.dendritic.cellna, type.1.T.helper.cellna, and type.2.T.helper.cellna were markedly different in GSE110169 (**Figure 6B**). Furthermore, the expression differences of m6A regulators between three gene clusters in GSE49454 and GSE110169 were evaluated. Sixteen m6A regulators were significantly different in GSE49454 (**Figure 6C**), and 21 m6A regulators were significantly different in GSE110169 (**Figure 6D**). Moreover, there were obvious differences in m6A score in the molecular subtypes (gene clusters and m6A clusters) of SLE in the GSE49454 (**Figures 6E, F**) and GSE110169 (**Figures 6G, H**) datasets.

### Consensus Clustering and Characteristics Analysis of ICI Clusters and Gene Clusters With Clinical Features

**Figure 7:**
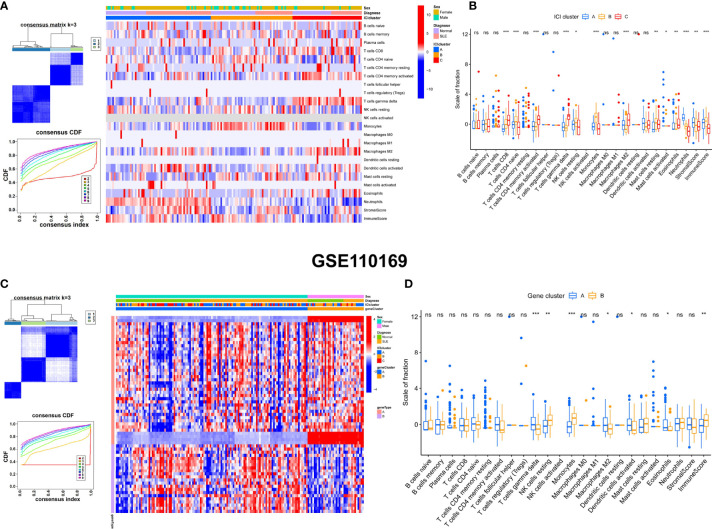
Construction of gene clusters and ICI clusters and comparison of the differences in immune cells in GSE110169. **(A)** The optimal cluster number was k = 3. CDF curves (k = 2–9). Heatmap of the expression patterns of genes (red: high expression; blue: low expression). The upper columns consist of sex, m6A clusters, and gene clusters of SLE. **(B)** Boxplot of differential immune cell infiltration between three ICI clusters (blue: ICI cluster A; yellow: ICI cluster B; red: ICI cluster C). **(C)** The consensus clustering number was k = 3. CDF curves (k = 2–9). A heatmap of the expression patterns of genes is shown. The upper columns consist of sex, ICI clusters, and gene clusters of SLE. **(D)** Fractions of infiltrated immune cells in two gene clusters of the GSE110169 cohort. *p < 0.05; **p < 0.01; ***p < 0.001; ns, not significant.

**Figure 8:**
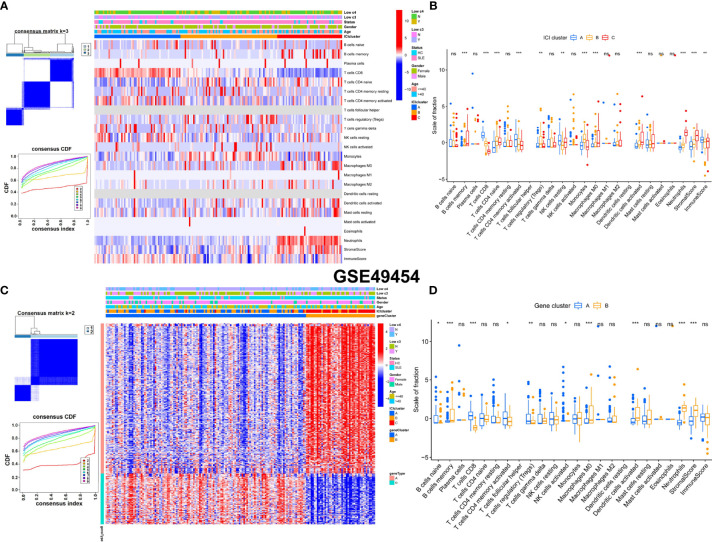
Construction of gene clusters and ICI clusters and comparison of the differences in immune cells in GSE49454. **(A)** The optimal cluster number was k = 3, CDF curves (k = 2–9). Heatmap of the expression patterns of genes (red: high expression; blue: low expression). The upper columns consist of age, sex, low c3, low c4, and ICI clusters of SLE. **(B)** Fractions of infiltrated immune cells in three ICI clusters in the GSE49454 cohort. **(C)** The consensus clustering number was k =2. CDF curves (k = 2–9). A heatmap of the expression patterns of genes is shown. The upper columns consist of age, sex, low c3, low c4, ICI clusters, and gene clusters of SLE. **(D)** Fractions of infiltrated immune cells in two gene clusters of the GSE49454 cohort. *p < 0.05; **p < 0.01; ***p < 0.001; ns, not significant.

Unsupervised consensus clustering was used to explore the molecular classification of SLE patients based on the expression patterns of immune cell infiltration (ICI). According to the relative change in the area under the CDF curve, the optimal number of clusters was determined to be three (k = 3). Hence, all SLE patients were categorized into three groups, which were termed ICI clusters 1–3. The differences in immune infiltration characteristics among the three m6A clusters and two gene clusters are shown by heatmaps and boxplots in GSE110169 (**Figures 7A–D**). The results revealed that naive CD4^+^ T cells, CD8^+^ T cells, activated memory CD4^+^ T cells, monocytes, gamma delta T cells, M2 macrophages, neutrophils, and immune scores were significantly different in the ICI clusters of GSE110169 (**Figures 7A, B** **)**. Gamma delta T cells and monocytes were significantly different in the gene clusters of GSE110169 (**Figures 7C, D** **)**. Consistently, to explore the differences in immune infiltration characteristics among three ICI clusters and two gene clusters, infiltrating immunocytes were also evaluated. Many immunocytes differed between the two patterns in GSE49454 (**Figures 8A–D**). Memory B cells, naive CD4^+^ T cells, CD8^+^ T cells, activated memory CD4^+^ T cells, monocytes, M0 macrophages, activated dendritic cells, neutrophils, stromal scores, and neutrophils were significantly different in the ICI clusters of GSE49454 (**Figures 8A, B** **)**. Memory B cells, CD8^+^ T cells, activated dendritic cells, stromal scores, and neutrophils were significantly different in the gene clusters of GSE49454 (**Figures 8C, D** **)**.

### Gene Modules, Univariate Cox Regression, and Functional Analysis of Immune Genes

**Figure 9:**
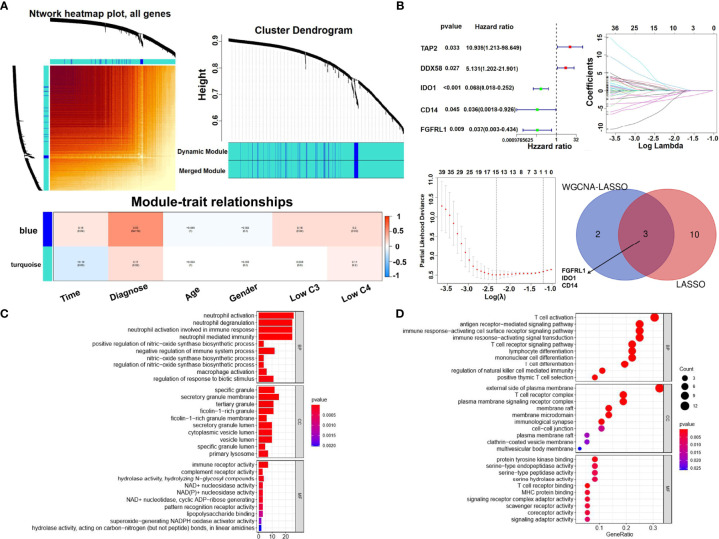
Gene modules, univariate Cox regression, and functional analysis of immune genes. **(A)** Gene modules identified by WGCNA. Correlation between gene modules and clinical features. Strongly correlated modules (|Cor> 0.5, p < 0.05) are marked with red frames. **(B)** Univariate Cox regression analysis of immune genes in the GSE49454 dataset. Partial likelihood deviance of different numbers of variables and LASSO coefficient profiles of immune genes in the GSE49454 dataset are shown. Venn diagram to identify key immune genes between WGCNA-LASSO and LASSO analysis. **(C)** Bar plot graph of GO enrichment based on the immune genes in the GSE49454 cohort (a longer bar means the more genes enriched, and an increasing depth of red means the differences were more obvious). **(D)** Bubble graph for KEGG pathway enrichment based on the immune genes in the GSE49454 cohort. (a larger bubble means that more genes were enriched, and an increasing depth of red means that the differences were more obvious).

A complete immune gene map was constructed. The related gene modules of different traits were identified by WGCNA. Two gene modules were identified to match different clinical characteristics, with the diagnosis closely associated with the genes in the blue module (**Figure 9A**). Univariate logistic regression was applied to identify immune genes associated with SLE, and five immune regulators (TAP2, DDX58, IDO1, CD14, and FGFRL1) were related to SLE (**Figure 9B**). Three key immune genes (FGFRL1, IDO1, and CD14) were selected by LASSO regression (**Figure 9B**). GO and KEGG enrichment analyses were used to investigate the biological pathways of the immune genes. The results revealed that they are mainly involved in neutrophil activation (**Figure 9C**). The biological processes involved in immune genes are closely related to T-cell activation pathways (**Figure 9D**).

### Biological Characteristics of Clustered Genes and Immune Genes in GSE110169

**Figure 10:**
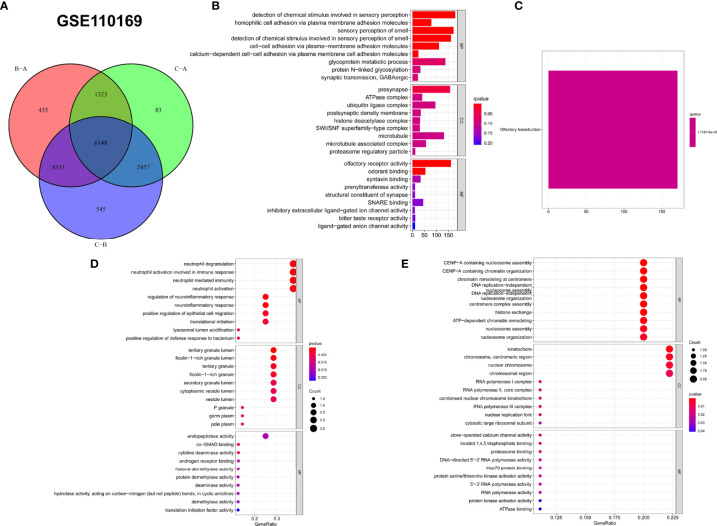
Functional analysis was performed on the common genes identified by gene clusters and the immune genes in the GSE110169 cohort. **(A)** Venn diagram of the common genes from three gene clusters between SLE and normal samples. **(B, C)** The GO and KEGG enrichment results were used to analyze the molecular functions, cellular components, and biological processes of the common genes of gene clusters, identified by bar plots. **(D**, **E)** Bubble graphs for GO and KEGG enrichment analysis of the immune genes in the GSE110169 cohort.

To screen for key genes, a total of 6,148 common genes were obtained from the intersection of clustered genes in GSE110169 (**Figure 10A**), and GO and KEGG enrichment analyses demonstrated that they mainly participated in the process of detection of chemical stimuli involved in sensory perception and olfactory transduction pathways (**Figures 10B, C** **)**. GO and KEGG pathway analyses were also used to evaluate the key biological pathways of immune genes in GSE110169. GO enrichment analysis showed that they are mainly involved in the processes of neutrophil degranulation and neutrophil activation during the immune response (**Figure 10D**). The biological pathways in which the immune genes take part were markedly related to CENP-A-containing nucleosome assembly, DNA replication-independent nucleosome assembly, etc. (**Figure 10E**).

### Univariate Cox Proportional Hazards Model for Diagnostic Prediction

**Figure 11:**
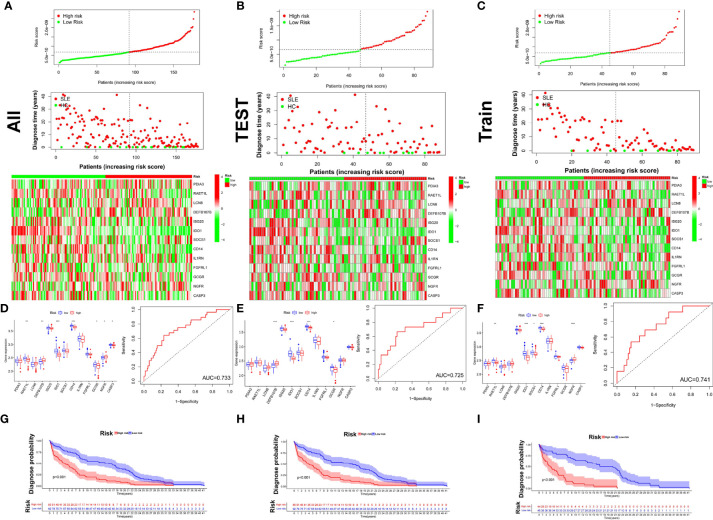
Diagnostic and time-dependent risk score analysis for the immune gene signature in SLE. **(A–C)** The signature risk score distribution, status of samples in the high- and low-risk groups, and heatmaps of the expression profiles of members in the 13 immune–gene signature in three sets [all **(A)**, test **(B)**, training **(C)**] of the GSE49454 dataset. **(D–F)** Boxplot of 13 diagnostic molecules between high- and low-risk patients and the ROC curve for diagnostic predictions in the three sets. **(G-I)** Kaplan–Meier curve between high- and low-risk patients. The validation sets using the test set and the entire set. *P < 0.05; **P < 0.01; ***p < 0.001.

We used univariate Cox proportional hazards model analysis to build a risk score model based on the median-risk score, which divided SLE patients and healthy samples into two groups: high risk and low risk in the GSE49454 dataset (**Figures 11A–C**). By LASSO regression analysis, 13 differentially expressed immune genes (PDIA3, RAET1L, LCN8, DEFB107B, ISG20, IDO1, SOCS1, CD14, IL1RN, FGFRL1, GCGR, NGFR, and CASP3) were screened out. Furthermore, we quantified the enrichment levels of the 13 genes in the two groups, and the results are shown in a heatmap (**Figures 11A–C** **)** and a boxplot (**Figures 11D–F**). The average AUC values for the sensitivity and specificity of the risk score and clinical factors in the three sets reached 0.733, 0.725, and 0.741. (**Figures 11D–F**). Kaplan–Meier curve analyses (**Figures 11G–I** **)** showed the diagnostic probability between high- and low-risk patients in the GSE49454 cohort (p < 0.001).

### Immune Cell Interactions and Characteristics of Immune Cells Between High- and Low-Risk Patients

**Figure 12:**
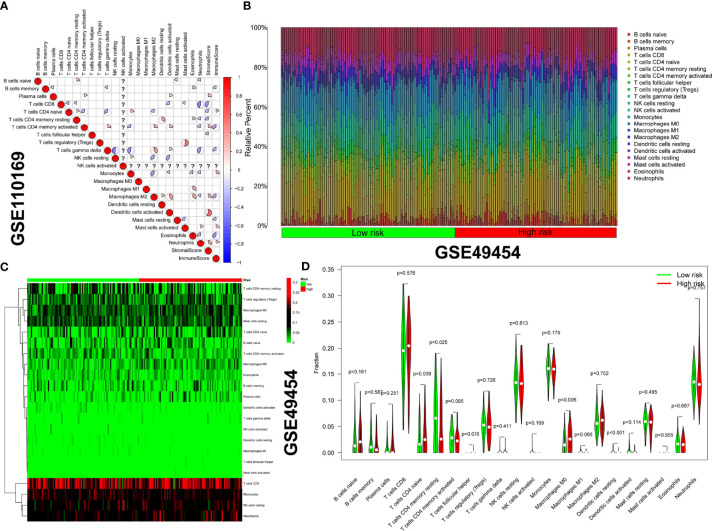
Correlation analysis of immune cells and difference in immune infiltration between high- and low-risk patients. **(A)** A correlation coefficient heatmap was generated to visualize the immune cell interaction (the color from red to blue represents positive and negative correlations, and the size of the pie graph represents the absolute correlation coefficient). **(B)** Relative proportions of immune infiltration in the high- and low-risk groups. **(C)** The heatmap illustrates immune infiltration in the high- and low-risk groups. **(D)** Violin plot of immune infiltration in the high- and low-risk groups.

The correlation coefficient heatmap provides an intuitive understanding of the state of immune cell interaction of GSE110169 (**Figure 12A**). CIBERSORT was used to calculate the immune cell expression of SLE subtypes in the GSE49454 dataset. The Wilcoxon test was used to compare the distribution of immune cells between high- and low-risk patients. We found that the differences in most immune cells were insignificant in the high-risk group compared with the low-risk group (**Figures 12B–D**). Only naive CD4^+^ T cells (p = 0.039) and M0 macrophages (p = 0.006) were higher in the low-risk group. In contrast, resting dendritic cells (p < 0.001) and resting memory CD4^+^ T cells (p = 0.025) were higher in the high-risk group (**Figure 12D**).

### Portfolio Analysis of the M6A Score and the ICI Score of the GSE49454 Dataset

**Figure 13:**
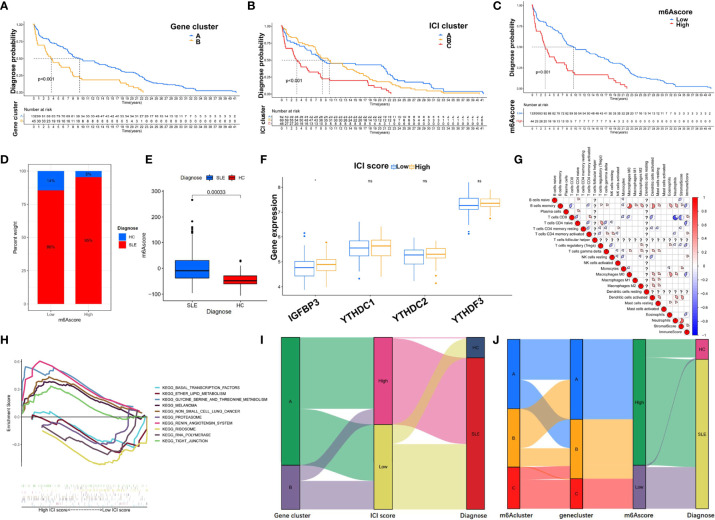
Portfolio analysis of the m6A and ICI scores of the GSE49454 dataset. **(A–C)** Kaplan–Meier curves for diagnosing the probability of m6A score, ICI score, and gene cluster groups (log-rank test: p < 0.001). **(D**, **E)** The proportion of m6A scores and diagnosis differences in m6A scores between the SLE (red) and HC (blue) groups in GSE49454. **(F)** Gene expression differences of four m6A regulators between high- and low-ICI score groups. **(G)** A correlation coefficient heatmap was generated to visualize the immune cell interaction. **(H)** GSEA plots showing the processes of glycine serine and threonine metabolism, renin–angiotensin system, tight junction, non-small cell lung cancer, and melanoma signaling pathways in the high ICI score group and the pathways of basal transcription factors, lipid metabolism, proteasome, RNA polymerase, and ribosome in the low ICI score group. **(I**, **J)** Alluvial diagram showing the relationship of gene cluster, ICI core, and diagnosis and the relationship of m6A cluster, gene cluster, m6A core, and diagnosis. *p < 0.05; ns, not significant.

The samples were unsupervised clustered using R packages. The graphs of unsupervised clustering of GSE49454 are shown in **Figures 5**, **8**. Significant differences in dependent clusters on diagnosis were revealed (log-rank test, p < 0.001) (**Figures 13A–C**). To examine the effectiveness of the m6A score in predicting clinical features, subjects were divided into high or low m6A score groups. The results showed that the number of SLE patients with high m6A scores was higher than that of patients with low m6A scores (**Figure 13D**). The comparison showed that the m6A scores of the SLE patients were higher than those of the healthy samples (**Figure 13E**). The Wilcoxon test showed that IGFBP3 was significantly overexpressed in the high ICI group (**Figure 13F**). The correlation coefficient heatmap revealed the immune cell interaction (**Figure 13G**). By GSEA, the remarkable enrichment of KEGG pathways delineated biological pathways or processes correlated with the ICI score in the GSE49454 cohort. Five representative KEGG pathways with high and low ICI scores were identified. The results are as follows: glycine serine and threonine metabolism, renin–angiotensin system, tight junction, non-small cell lung cancer, and melanoma signaling pathways were associated with a high ICI score, while enrichment plots demonstrated that basal transcription factors, lipid metabolism, proteasome, RNA polymerase, and ribosome pathway were associated with a low ICI score (**Figure 13H**). The relationships of gene cluster, ICI score, diagnosis, and the relationship of m6A cluster, gene cluster, m6A score, and diagnosis were also shown (**Figures 13I, J** **)**.

### Protein Structure Prediction Comparison and Prediction of Drug Targets of Key Genes

**Figure 14:**
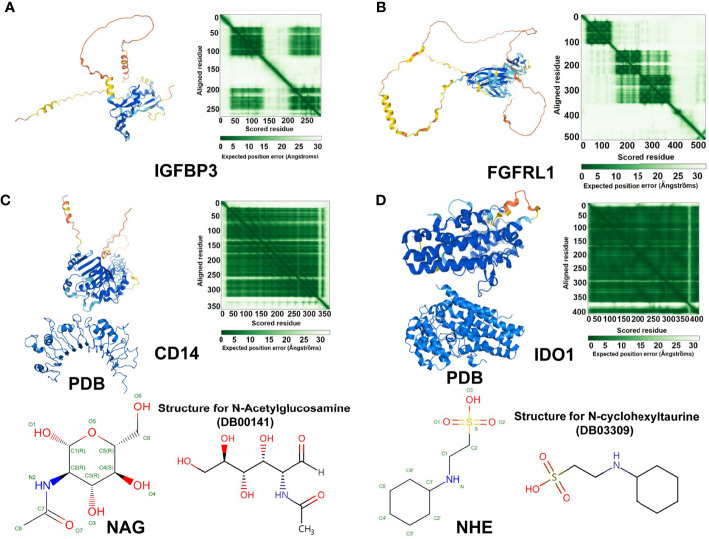
Protein structure prediction comparison and drug targets of IGFBP3, FGFRL1, CD14, and IDO1. AlphaFold protein structure predictions of **(A)** IGFBP3, **(B)** FGFRL1, **(C)** CD14, and **(D)** IDO1. AlphaFold produces a per-residue confidence score (pLDDT) between 0 and 100. Some regions below 50 pLDDT may be unstructured in isolation. The shade of green indicates the expected distance error in Ångströms. The dark green module corresponds to the highlighted region. Dark green indicates good error, and light green indicates bad error. **(C)** Representative CD14 structure in the PDB database, which contains experimental structural data. In addition, the drug-target complex N-acetylglucosamine (NAG) bound to its directly interacting ligands. **(D)** PDB structure chain showing IDO1 and the drug-target complex: N-cyclohexyltaurine (NHE) bound to its directly interacting ligands. We thank all members of the laboratory for useful discussions.

The AlphaFold Protein Structure Database (https://alphafold.ebi.ac.uk/) was used to predict the protein structures of IGFBP3, FGFRL1, CD14, and IDO1 (**Figures 14A–D**). We also compared the representative CD14 and IDO1 structures in the Protein Data Bank (PDB) database, which contains experimental structural data. IGFBP3 and FGFFRL1 were not included in PDB. We screened the drug-target complex from DrugBank (https://go.drugbank.com/) according to each target’s directly interacting ligands.

## Discussion

SLE is a multifactorial and complex autoimmune disease that is characterized by the deposition of immune complexes and the production of autoantibodies; it also features various cellular and molecular aberrations and predominantly affects female individuals (33). Genetic, hormonal, environmental, and other factors trigger SLE, causing multivisceral dysfunction. To date, the pathogenesis of SLE remains unclear (34). Epigenetic factors, especially m6A modification, play a key role in the process of SLE. However, only sporadic studies have evaluated m6A modifications. For example, the findings of Luo et al. suggested that decreased YTHDF2 was related to the disease activity of SLE, and ALKBH5 may be involved in the pathogenesis of SLE (35, 36). Li et al. (37) summarized the mechanisms of m6A modification in gene expression regulation and immune response (such as modulating pre-mRNA splicing, RNA structure, mRNA stability, pri-miRNA, and translation). The authors speculated that m6A modification may take part in the initiation and progression of SLE. Therefore, it is necessary to further explore the potential research value of this aspect.

In this study, compared with the healthy control group, by analyzing four datasets, we identified 19 key m6A regulators (p < 0.05). We then investigated the biological functions of these m6A modulators by GO and KEGG analysis, and the results revealed that these regulators are significantly associated with changes in the regulation of mRNA metabolic processes, mRNA processing, etc. KEGG pathway analyses indicated that these genes were mainly enriched in the regulation of insulin-like growth factor (IGF) activity by the insulin-like growth factor binding protein (IGFBP) pathway. Moreover, the gene–gene network of m6A regulators and the TF–miRNA network of key m6A risk regulators were constructed. These regulators could contribute to promoting SLE therapy. Furthermore, immune genes associated with SLE were identified using WGCNA in this study. Immune genes in the high diagnostic significance module were selected as central genes. We also obtained key m6A-immune genes associated with the diagnosis of SLE patients by univariate and multivariate Cox regression analyses and the Kaplan–Meier method. By GO and KEGG analysis, we further analyzed the functions of the common differentially expressed genes identified by gene clusters and the immune genes between the high- and low-risk groups. ROC analyses were applied to explore the sensitivity and specificity of clinical factors and risk score and IGFBP3 in different datasets for SLE diagnosis. In addition, another six independent datasets (GSE50772, GSE81622, GSE122459, GSE20864, GSE39088, and GSE156751) were used for external validation to identify the stable differential expression of IGFBP3 between SLE and healthy samples. Ultimately, three key immune genes (FGFRL1, IDO1, and CD14) and one m6A regulator (IGFBP3) were screened. The results demonstrated that these genes screened in the present study could act as promising biomarkers for the diagnosis and treatment of SLE.

CD14 (CD14 molecule) is preferentially expressed on monocytes and macrophages and mediates the innate immune response to viruses. It has been identified as a candidate target for treating severe acute respiratory syndrome coronavirus 2 (SARS*-*CoV*-*2*)*-infected patients by reducing or suppressing severe inflammatory responses (38, 39). It is mainly involved in the Toll comparison pathway and the innate immune system. The CD14 (C-159T) polymorphism was related to increased susceptibility to SLE and could be a promising biomarker for the diagnosis of lupus nephritis (40). Moreover, a study demonstrated that urinary CD14 mononuclear cells could serve as a biomarker for lupus nephritis (LN) (41). The enzyme indoleamine 2,3-dioxygenase 1 (IDO1) is a heme enzyme that regulates immune responses to arrest inflammation (42). IDO1 maintains homeostasis by preventing autoimmune or immunopathology from participating in peripheral immune tolerance (43). Furthermore, regulatory abnormalities of IDO1 have been demonstrated in patients with SLE (44). Low IDO1 expression in human induced pluripotent stem cells (hiPSCs) of SLE can cause abnormal activation of the immune response (45). IDO has been suggested to play a key role in a variety of autoimmune diseases, including MRL/lpr mouse models of lupus-like diseases, and in another study, IDO1 protein and IDO total enzyme activity were significantly increased in lupus-prone B6.Nba2 mice compared to B6 controls (46). Fibroblast growth factor receptor-like 1 (FGFRL1) belongs to one of the fibroblast growth factor receptor (FGFR) families and has a negative effect on cell proliferation. FGFRL1 has been reported in cancer but not in SLE (47, 48).

Insulin-like growth factor binding protein 3 (IGFBP3) is a protein that regulates the growth and proliferation of somatic cells. Insulin-like growth factors (IGFs) are important growth-promoting factors. IGFBP-3 plays a key role in the secretion and action of growth hormone (49). Most IGF molecules interact with members of the IGF-binding protein (IGFBP) family in blood flow and local tissues (50). Free insulin-like growth factor-1 (IGF1) has a positive metabolic effect in SLE, and the level of IGF1 decreases appropriately with increasing age. It may indirectly inhibit the immune response by downregulating T- and B-cell activity (51). Overexpression of IGF-I and IGFBP2 in glomeruli may play important roles in renal function and morphological changes in MRL/LPR mice (52). IGFBP2 has been identified as a biomarker of SLE and lupus nephritis (53–55). IGFBP3 has also been shown to play a major role in maintaining a healthy immune system, supporting the maintenance and development of naive CD8+ T cells (56). Most studies have shown that IGFBPs have great potential as biomarkers in autoimmune diseases (57).

Molecular analysis of SLE demonstrated that many molecular components are significantly related to the immune response or diagnosis of SLE. Changes in these molecules in SLE may interfere with the communication between immune cells, affecting the balance of immune tolerance and immune activity. We also performed a detailed characterization of m6A or ICI profiles, and the m6A-immune patterns offer a glimmer of hope for patient-specific individualized therapy. Therefore, we also established models to evaluate the impacts of m6A immunity on SLE. Some studies have demonstrated that epigenetic and immune disorders are involved in SLE progression (37, 58–61). M6A regulators could serve as biomarkers of diseases, and the dysfunction of immune cells might protect against disease by therapeutic intervention. Herein, we mainly focused on the characterization of m6A immunity in SLE, which plays a major role in mediating the immune system. Our study analyzed a number of samples from different datasets and categorized the samples into m6A immune-related subgroups. Thus, according to the m6A and ICI gene clusters, we first obtained the m6A-immune subgroups related to the clinical characteristics. These results indicated that the m6A and ICI scores are a useful biological diagnostic method for evaluating the potential of immunotherapy. Considering the robustness of the above model, we combined the samples to generate a global prediction model containing multiple variables. We found differences in immune infiltration characteristics among m6A, ICI clusters, and gene clusters. Many immunocytes differ among these patterns. Based on the findings of our study, the involvement of m6A or ICI gene clusters in the immune response was associated with diagnosis and might trigger systemic immune responses and resistance to immunotherapy. Thus, we speculated that the m6A-immune phenotypes could help predict the reaction to immune therapy.

Genomic studies have also shown that SLE is an autoimmune disease with a high degree of immune cell infiltration (62, 63). For example, kidney-infiltrating T cells (KITs), as activated effector cells, may cause organ failure and tissue damage (64). CD4^+^Foxp3^+^IL-17A^+^ cell infiltration was found in renal biopsy specimens of active lupus nephritis (65). Moreover, urinary CD11c+ macrophages expressed proinflammatory cytokines (IL-6 and IL-1) and resembled infiltrated monocytes (66). Yoshikawa et al. revealed that CXCR5− and CXCR3+ B cells were elevated and involved in B-cell infiltration into tissues and the inflammatory pathogenesis in SLE (67). In this study, we also used GSVA to explore the molecular pathways and underlying mechanisms in B-cell subsets between SLE patients and healthy controls. The results showed that neuroactive ligand receptor interactions, olfactory transduction, etc. pathways were positively correlated with SLE. DNA replication, RNA polymerase, neurotrophin signaling, spliceosome, etc. pathways were negatively correlated with SLE. Furthermore, immune cell infiltration has also been detected in lupus-prone mice. For example, IL-22 in renal epithelial cells of MRL/lpr mice has been found to bind to IL-22R to activate the STAT3 signaling pathway, enhance chemokine secretion, and promote macrophage infiltration into the kidney, exacerbating lupus nephritis (68). Type I interferon (IFN) signatures and increased immune cell infiltration were consistent with the severity of lupus in the kidneys of Aim2−/− mice (69). Immune cell infiltration in SLE is a complex and variable process that is actively involved in the regulation of inflammatory responses to promote or suppress the disease process. In the m6A and ICI gene clusters, there appears to be an association between immune cell infiltration and response, which is a favorable diagnosis. Therefore, we hypothesized that m6A-immune therapy might be beneficial to patients with SLE, which suggests that the different characteristics of m6A and ICI gene clusters obtained in our study may help in the development of more accurate immune therapy.

In the current study, we attempted to identify m6A targets for SLE and to explore the role of m6A-immune interaction models in SLE. The results in our study were obtained from the bioinformatic analysis only. Hence, more studies are needed to confirm the roles of m6A immunity in different pathways.

## Conclusions

Overall, we conducted a comprehensive analysis of the m6A and ICI profiles of SLE. Our study provides clear information on the regulation of the m6A immune response in SLE. We discovered the links between different patterns of m6A immunity, heterogeneity of m6A immunity, and potential treatment targets. IGFBP3 and immune genes (CD14 and IDO1) screened in the present study could serve as promising targets for the treatment of SLE. This study has significant clinical significance for the comprehensive evaluation of m6A immune patterns, which may provide a new direction for the understanding of SLE. It would also help in the selection of optimal strategies for personalized immunotherapy.

## Data Availability Statement

The datasets presented in this study can be found in online repositories. The names of the repository/repositories and accession number(s) can be found in the article/**Supplementary Material**.

## Author Contributions

XZ: conception and design, data analysis, and writing—original draft. LG and JW: formal analysis and validation. BN, ZS, XH, and ZR: reviewing and revising the manuscript, guidance for manuscript preparation, and final approval of the manuscript. YY: reviewing the manuscript and funding acquisition and final approval of the manuscript. All authors contributed to the article and approved the submitted version.

## Funding

This study was supported by the National Natural Science Foundation of China (No. 81673058) and Natural Science Foundation of Chongqing (cstc2021jcyj-msxm3786).

## Conflict of Interest

The authors declare that the research was conducted in the absence of any commercial or financial relationships that could be construed as a potential conflict of interest.

## Publisher’s Note

All claims expressed in this article are solely those of the authors and do not necessarily represent those of their affiliated organizations, or those of the publisher, the editors and the reviewers. Any product that may be evaluated in this article, or claim that may be made by its manufacturer, is not guaranteed or endorsed by the publisher.

## Supplementary Material

Supplementary Table 1 Datas of the gene expression of GSE49454.

Supplementary Table 2 Clinical information of GSE49454 dataset.

Supplementary Table 3 Datas of the gene expression of GSE61635.

Supplementary Table 4 Datas of the gene expression of GSE110169.

Supplementary Table 5 Clinical information of GSE110169 dataset.

Supplementary Table 6 Datas of the gene expression of GSE72509.

Supplementary Table 7 Datas of the gene expression of GSE50772.

Supplementary Table 8 Datas of the gene expression of GSE81622.

Supplementary Table 9 Datas of the gene expression of GSE122459.

Supplementary Table 10 Datas of the gene expression of GSE20864.

Supplementary Table 11 Datas of the gene expression of GSE39088.

Supplementary Table 12 Datas of the gene expression of GSE156751.
