# Supplementary material for: Exploration of Potential Integrated Models of N6-Methyladenosine Immunity in Systemic Lupus Erythematosus by Bioinformatic Analyses
Source: Front Immunol. 2022 Feb 7;12:752736. doi: 10.3389/fimmu.2021.752736 (PMC8859446; doi:10.3389/fimmu.2021.752736)
Supplement: Supplementary file 13 [file DataSheet_2.doc]

**Supplementary Figure**


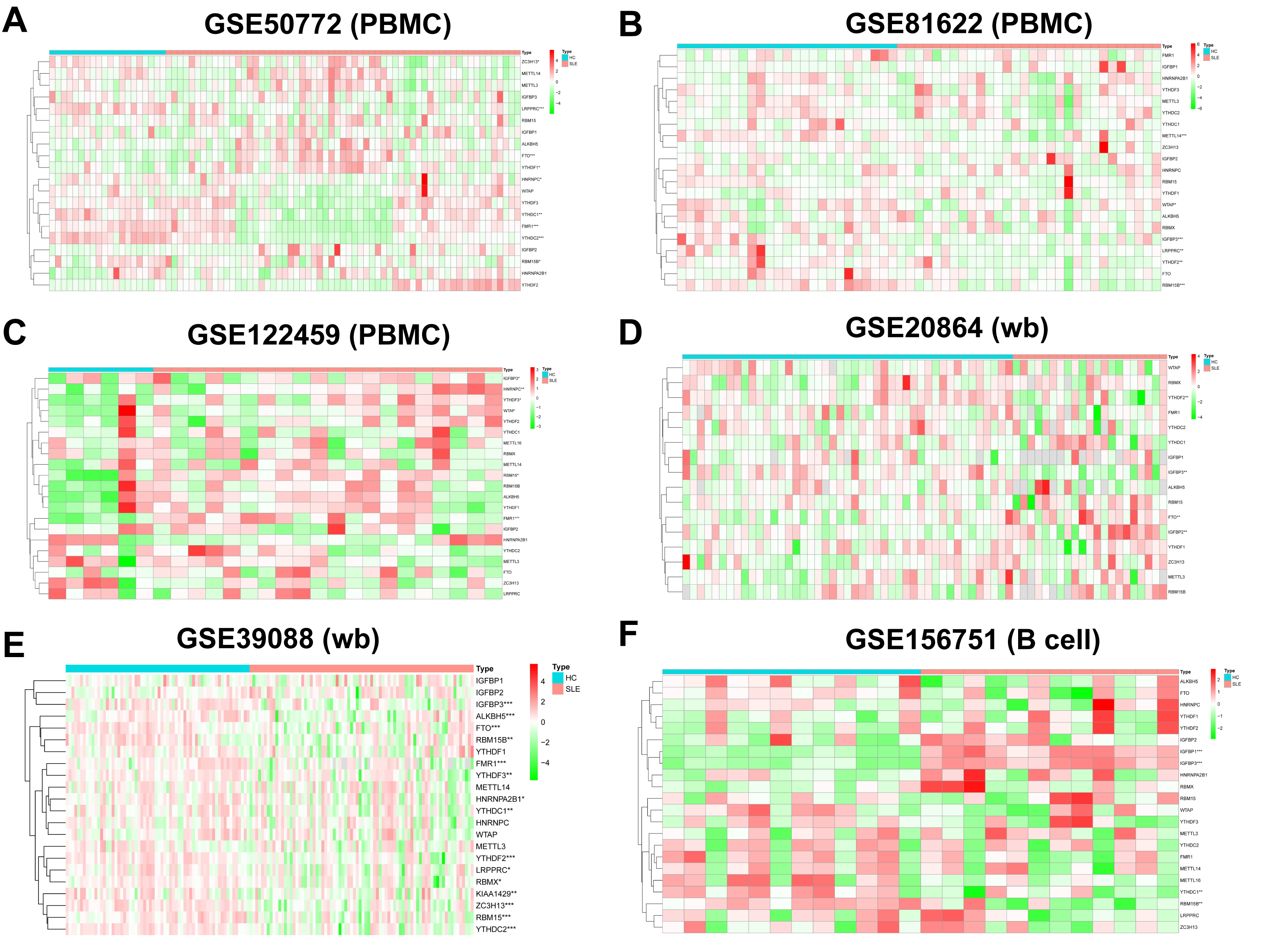


**FIGURE S1** | Validation of the key m6A regulators. **(A-F)** The differentially expressed m6A regulators was evaluated in **(A)** GSE50772, **(B)** GSE81622 **(C)** GSE122459, **(D)** GSE20864, **(E)** GSE39088 and **(F)** GSE156751 datasets, respectively. Green: low expression level; red: high expression level. *P < 0.05, **P < 0.01, ***p<0.001.

**Supplementary Table**

**Supplementary Table 13. Data cohort characteristics**

| Datasets | SLE (N) | Normal (N) |
| --- | --- | --- |
| GSE49454 (whole blood) | 157 | 20 |
| GSE61635 (whole blood) | 99 | 30 |
| GSE110169 (whole blood) | 82 | 77 |
| GSE72509 (whole blood) | 99 | 18 |
| GSE50772(PBMCs) | 61 | 20 |
| GSE81622 (PBMCs) | 30 | 25 |
| GSE122459 (PBMCs) | 20 | 6 |
| GSE20864 (whole blood) | 21 | 45 |
| GSE39088 (whole blood) | 78 | 64 |
| GSE156751 (B cells) | 12 | 12 |
| GSE80183 (whole blood) | 8 | 8 |

*SLE: Systemic Lupus Erythematosus.*
